# A Proteomic Investigation of Soluble Olfactory Proteins in *Anopheles gambiae*

**DOI:** 10.1371/journal.pone.0075162

**Published:** 2013-11-25

**Authors:** Guido Mastrobuoni, Huili Qiao, Immacolata Iovinella, Simona Sagona, Alberto Niccolini, Francesca Boscaro, Beniamino Caputo, Marta R. Orejuela, Alessandra della Torre, Stefan Kempa, Antonio Felicioli, Paolo Pelosi, Gloriano Moneti, Francesca Romana Dani

**Affiliations:** 1 Integrative Proteomics and Metabolomics, Max-Delbrück-Center for Molecular Medicine, Berlin, Germany; 2 China-UK-NYNU-RRes Joint Libratory of Insect Biology, Nanyang Normal University, Nanyang, China; 3 Department of Agriculture, Food and Environment, Università di Pisa, Pisa, Italy; 4 Department of Veterinary Sciences, Università di Pisa, Pisa, Italy; 5 CISM, Mass Spectrometry Centre, Università di Firenze, Firenze, Italy; 6 Department of Public Health and Infectious Diseases, Università “La Sapienza”, Roma, Italy; 7 Systems Biology of Gene Regulatory Elements, Max-Delbrück-Center for Molecular Medicine, Berlin, Germany; 8 Biology Department, Università di Firenze, Firenze, Italy; University of California Davis, United States of America

## Abstract

Odorant-binding proteins (OBPs) and chemosensory proteins (CSPs) are small soluble polypeptides that bind semiochemicals in the lymph of insect chemosensilla. In the genome of *Anopheles gambiae*, 66 genes encode OBPs and 8 encode CSPs. Here we monitored their expression through classical proteomics (2D gel-MS analysis) and a shotgun approach. The latter method proved much more sensitive and therefore more suitable for tiny biological samples as mosquitoes antennae and eggs. Females express a larger number and higher quantities of OBPs in their antennae than males (24 *vs* 19). OBP9 is the most abundant in the antennae of both sexes, as well as in larvae, pupae and eggs. Of the 8 CSPs, 4 were detected in antennae, while SAP3 was the only one expressed in larvae. Our proteomic results are in fairly good agreement with data of RNA expression reported in the literature, except for OBP4 and OBP5, that we could not identify in our analysis, nor could we detect in Western Blot experiments. The relatively limited number of soluble olfactory proteins expressed at relatively high levels in mosquitoes makes further studies on the coding of chemical messages at the OBP level more accessible, providing for few specific targets. Identification of such proteins in *Anopheles gambiae* might facilitate future studies on host finding behavior in this important disease vector.

## Introduction

Mosquitoes are vectors of several diseases affecting about one hundred million people worldwide and killing more than a million, mostly in tropical areas [1]. In the absence of protective vaccines, as is the case of malaria and dengue fever, at present transmission of the pathogens to humans is avoided using bed nets, and mosquitoes populations are controlled mainly with insecticide-based strategies. Although this last approaches may be very efficient, they are also unsafe for human health and for the environment. Moreover, insects can rapidly develop resistance to insecticides, thus continuously requiring the design and the use of new generations of chemicals. Therefore, alternative approaches to fight mosquitoes are strongly needed. A promising strategy is to target the chemical communication system of mosquitoes with the aim of developing efficient repellents that might interfere with the olfactory system and disrupt the perception of chemical messages, such as those that allow host localization and choice. In this respect, an interesting approach is suggested by the observation that high levels of carbon dioxide can disorient mosquitoes [2]. However the commercially available synthetic products have recently raised some concern for human health [3], prompting a wide research on alternative mosquito repellents. Such investigation requires a detailed knowledge of the mosquito's chemoreception system at the molecular level in order to understand which chemical messages are important for the insect biology and the behavioural responses they induce.

Two classes of proteins are directly involved in the perception and recognition of chemical stimuli, membrane-bound olfactory (OR) and gustatory (GR) receptors and soluble Odorant-Binding Proteins (OBPs) [4].

In particular, recent research has provided several pieces of evidence on the specific involvement of OBPs in the detection and discrimination of chemical messages in insects [5]–[10]. Therefore, a study on the structure and properties of the different OBPs could represent a strong basis for understanding the olfactory code in a given species and help designing new compounds that may be effective in population control.

*Anopheles gambiae* is the main malaria vector in sub-Saharan Africa. The genome of the species [11] has provided valuable information for the study of chemoreception proteins. It contains 79 genes encoding olfactory receptors and 76 encoding gustatory receptors [12]–[14]. These genes have been expressed in different systems and their specificities in recognising chemical stimuli have been analysed [15], [16].

While there is little doubt that all (or at least most of) the membrane-bound receptors classified as olfactory and gustatory are involved in the perception of external chemical stimuli, with OBPs the picture is much more complex. In fact, this large family of proteins comprises members that may perform different functions, indirectly related or even completely unrelated to olfaction and taste, such as transport of semiochemicals in reproductive organs [17] or binding of biogenic amines [18].

The genome of *An. gambiae* contains 66 genes encoding proteins that have been classified as OBPs solely on the basis of sequence similarity [14], [19]. This number is very close to that of olfactory receptors and at the beginning suggested the idea that a one to one relationship could exist between members of the two families of proteins. However, this view proved to be too simplistic and the actual situation is much more complex. Only 33 of such genes encode so-called “classic OBPs”, whose signature is a conserved pattern of six cysteines, linked to each other by disulfide bonds in a specific fashion (1–3, 2–5, 4–6) [20], [21]. The relative positions and the pairing of the six cysteines are conserved across all Orders of insects, from locusts and aphids to Coleoptera and Diptera. In addition, there are 19 longer OBPs in *An. gambiae*, containing a larger number of cysteines and therefore called C-plus OBPs. Their sequences still present a “classic” core with additional polypeptide segments [22]. A third group of 14 proteins includes outliers and is classified under the name of “atypical OBPs”. Among these, some are referred to as “tandem OBPs”, containing two “classic OBP” sequences connected by few amino acids. These proteins, that occur in the saliva of mosquitoes, are probably not involved in chemoreception, on the basis of a recent report showing that one member binds biogenic amines and mediates antiinflammatory processes [18].

The picture is still more complex with the other family of soluble proteins of the chemoreception system, the Chemosensory Proteins (CSPs) [4], [23], [24]. In fact, several members of this group are expressed in non-sensory organs and some are involved in different functions, such as development and differentiation [25]–[31]. These polypeptides are shorter than OBPs (100–120 residues) and present only 4 cysteines paired in non-interlocked fashion [32]. In *An. gambiae* only 8 genes encoding such proteins have been identified, and reported alternatively as CSPs or SAPs (Sensory Appendage Proteins) [14], [33], [34].

Because of such complex picture, it is important to identify which OBPs and CSPs are expressed in antennae and other sensory organs, such as mouth parts and tarsi, being these proteins more likely involved in the perception of semiochemicals.

Using microarrays, Biessmann and coworkers [14], found that the most abundantly expressed OBPs in female antennae are in the order: 5, 48, 1, 17, 9, 47, 3, 7, 4 and 20. All of them are classic OBPs with the exception of C-plus OBP47 and OBP48. Most of these proteins are expressed at higher levels in female antennae than in males', while OBPs 5 and 9 are more abundant in males. In the same study, several genes are reported to be down-regulated in the female antennae after a blood meal, with the exception of OBP9, whose level of mRNA greatly increased after a blood meal. Among the CSPs, only the RNAs encoding the three SAPs were detected in the antennae of *An. gambiae* [14]. A more recent report, aimed at characterising trascriptome profile in chemosensory tissues, partially confirmed the data discussed above [35]. In larvae and pupae, several OBPs were detected using microarray and PCR analysis, the most abundant being #9, 1, 17, 48, 3, 4, 5 [14]. A proteomic investigation reported the presence of OBPs #1, 11, 13, 34, 35, 36, 37 and 44 on the egg shells [36].

There is a large amount of information regarding the three-dimensional structure of *An. gambiae* OBPs, with respect to similar proteins in other species. In fact, the folding of classic OBPs # 1, 4, 7, 20, 22a, and C-plus OBP47 has been solved [22], [37]–[42]. Moreover, OBP1 and OBP4 have been co-crystallized [42], supporting a previous report of functional interactions between these two proteins [43].

Here, we adopted a proteomic approach to identify OBPs and CSPs that are expressed in the antennae of *An. gambiae* males and females, as well as in pre-adult stages. The results show that only about one third of the genes encoding OBPs and half of those encoding CSPs are expressed at the protein level in antennae with a strong sexual dimorphism, while in pre-adult stages OBP9 is the by far the most abundant.

## Materials and Methods

### Ethics statement

This study was approved by the Ethical Committee of the University of Pisa, N. 12498. The rabbits were bled under anaesthetic from the heart.

### Reagents

All enzymes, unless otherwise stated, were from New England Biolabs. Oligonucleotides were custom synthesized at Eurofins MWG GmbH, Ebersberg, Germany. All other chemicals, unless otherwise stated, were purchased from Sigma-Aldrich and were of reagent grade.

### Preparation of extracts

*Anopheles gambiae* were reared at the Department of Public Health of the University “La Sapienza”, Roma, Italy, from a colony named GA-CAM-ST originated from the progeny of females collected in Cameroon and belonging to the molecular form M (standard with regard to the chromosomal inversions, [44]). All adult specimens were 2 days old and were fed only with 0.5% sugar solution. Females and males were segregated in different cages soon after emergence to keep them virgin. Specimens were killed by freezing at −20°C and then transferred at −80°C.

For 2D gel separation, the antennae of 1,110 male individuals were used. For shotgun proteomic experiments we used in total the antennae from 600 individuals of each sex to perform three sets of analysis, each in triplicate. Antennae were crushed in a mortar under liquid nitrogen and extracted with 0.1% trifluoroacetic acid. The extracts were centrifuged at 19,000× g for 40 min at 4°C and the supernatants were concentrated to 50 µL by centrifugal evaporation.

100 fourth instar larvae, or 100 pupae, or 100 eggs of *An. gambiae* were homogenised in 500 µL of 0,1% aqueous TFA by grinding in a mortar followed by sonication, and centrifuged at 19,000× g for 40 min at 4°C.

### Two-dimentional electrophoresis and identification of protein spots

Along with our previously described protocol [45], the extracts were concentrated to 50 µL and then diluted to 250 µL with a buffer containing 7 M urea, 2 M thiourea, 2% (w/v) CHAPS, 1% (v/v) IPG buffer (GE-Healthcare) and 60 mM of Dithiothreitol (DTT). The samples were loaded by rehydration for 11.5 hours in IPG strips (pH 3–11, 7 cm). Isoelectrofocusing was performed with an Ettan IPG Phor III system (GE-Healthcare) using the following conditions: 50 V (2 hours), 100 V (2 hours), 500 V (2 hours), 1000 V (2 hours), 6000 V (1.5 hours). Strips were equilibrated for 15 minutes in a TrisHCl 1.5M pH 8.8 solution containing glycerol 29.3%, urea 6 M, SDS 2% (w/v), DTT 1% and then for further 15 minutes in a Tris-HCl 1.5M pH 8.8 solution, containing glycerol 29.3%, urea 6M, SDS 2% and Iodoacetamide 2.5%.

Gels were stained using Brilliant Blue G-Colloidal Concentrate (Sigma). The excised spots were subjected to tryptic digestion and nano HPLC-ESI Orbitrap analyses. The acquired MS and MS/MS data were searched with Proteome Discoverer 1.2 (Thermo Fisher) using SEQUEST as the search algorithm against a database created by merging the sequences of the peptides predicted from *An. gambiae* genome [11] (Anopheles_gambiae.AgamP3.48.pep.all.fa.gz, and Anopheles_gambiae.AgamP3.48.pep.abinitio.fa.gz downloaded at http://www.ensembl.org/info/data/download.html) with the entries related to *Anopheles* in UniProtKB. Searches were performed allowing up to three missed cleavage sites, 10 ppm of tolerance for the monoisotopic precursor ion and 0.5 mass unit for monoisotopic fragment ions and carbamidomethylation of cysteine and oxidation of methionine as variable modifications. False discovery rate was set at 1%.

### Shotgun experiments

#### Antennae

Samples for shotgun experiments were resuspended in 200 µL of urea containing buffer (8 M Urea, 100 mM TrisHCl, pH 8.5). Based on Bradford colorimetric assay, the samples of female and male antennal extracts contained 80 and 200 µg of total protein, respectively. Reduction of disulfide bridges and alkylation was performed by treating samples with 2 mM DTT (30 minute at 25°C), followed by 11 mM iodoacetamide (20 minutes at room temperature in the dark). LysC digestion was then performed by incubating the samples with LysC (Wako) in a ratio 1∶40 (w/w) under gentle shaking at 30°C. The digestion products were diluted 3 times with 50 mM ammonium bicarbonate and incubated with 10 µL of immobilized trypsin (Applied Biosystems) for 4 hours under rotation at 30°C.

Fifteen 15 µg of each resulting peptide mixture were then desalted on Stage Tip [46] and the eluates dried and reconstituted to 50 µL in 0.5% acetic acid. Fractions containing 7 µg of protein were injected.

The extract was analysed on three sets of analyses, each performed in triplicates on a LC-MS/MS system (Eksigent nano Liquid Chromagrapher coupled to a Linear Trap Quadrupole -Orbitrap Velos (Thermo)), on a C18 (75 µm i.d.×15 cm, 1.8 µm, 100 Å) column at 250 nL/min using a 155 or 255 minutes gradient ranging from 5% to 60% of solvent B (solvent A = 5% acetonitrile, 0.1% formic acid; solvent B 80% acetonitrile, 0.1% formic acid). The nanospray source was operated with a spray voltage of 2.1 kV and ion transfer tube temperature of 275°C. Data were acquired in data dependent mode, with one survey MS scan in the Orbitrap mass analyzer (resolution 60,000 at m/z 400) followed by up to 20 MS/MS in the ion trap on the most intense ions (intensity threshold = 750 counts). Once selected for fragmentation, ions were excluded from further selection for 30 seconds, in order to increase new sequencing events. Raw data were analyzed using the MaxQuant proteomics pipeline (v1.2.2.5) and the ANDROMEDA search engine [47] against the database described above. Carbamidomethylation of cysteines was chosen as fixed modification, oxidation of methionine and acetylation of N-terminus were chosen as variable modifications. The search engine peptide assignments were filtered at a False Discovery Rate <1% and the feature “match between runs” was not enabled; other parameters were left as default.

For each set of analysis, relative abundance of proteins was estimated using the “Intensity” values as produced by MaxQuant software [47], normalised on the total intensity signal. The results of the three sets were averaged.

### PFAM enrichment analysis

Each identified protein was assigned to its Protein family (Pfam) [48] and Pfam were analysed for differential expression between male and female antennae. Proteins were considered to be expressed in only one sex if identification was based on more than 2 peptides and no peptides were identified in the other sex. Proteins were considered overexpressed if the ratio of intensity values between the two sexes was greater than three. The Pfam enrichment analysis was performed using custom R scripts (available on demand). For each individual Pfam id associated to proteins overexpressed or found in only sex, a Fisher exact test was performed over the total set of proteins. Results were filtered with an alpha = 0.05.

#### Eggs

Eggs extract was freeze-dried, redissolved in 40 µL of 10 mM DTT in100 mM ammonium bicarbonate and incubated at 56°C for 45 min. Then, 40 µL of 55 mM iodoacetoamide were added and the mixture was incubated at room temperature for 30 min in the dark. Digestion was performed by addition of 2 µL of 0.1 µg/µL trypsin and incubation overnight at 37°C. Digestion was blocked by 10% TFA to pH 2.5. Aliquots of 25 µL of the resulting peptide mixture were then desalted on three Stage Tips (Rappsilber et al., 2007); eluates were pooled, dried and then reconstituted to 15 µL in 0.5% acetic acid. Peptide solution was analysed in triplicates (1 µL) on a Ultimate 3000 HPLC (Dionex, San Donato Milanese, Milano, Italy) coupled with an Linear Trap Quadrupole Orbitrap mass spectrometer (Thermo Fisher, Bremen, Germany) using a C18 (75 µm i.d.×15 cm, 1.8 µm, 100 Å) column at a 250 nL/min flow, using a 144 min gradient ranging from 5% to 90% of solvent B (solvent A = 5% acetonitrile, 0.1% formic acid; solvent B 80% acetonitrile, 0.1% formic acid). The nanospray source was operated with a spray voltage of 2.0 kV and ion transfer tube temperature of 275°C. Data were acquired in data dependent mode, with one survey MS scan in the Orbitrap mass analyzer (resolution 15,000 at m/z 400) followed by up to 3 MS/MS in the ion trap on the most intense ions. The acquired MS and MS/MS data were searched with Proteome Discoverer 1.2 (Thermo Fisher) using SEQUEST as the search algorithm, as described above.

### RNA extraction and cDNA synthesis

Total RNA was extracted with the TRI® Reagent (Sigma), following the manufacturer's protocol. cDNA was prepared from total RNA by reverse transcription, using 200 units of SuperScriptTM III Reverse Transcriptase (Invitrogen) and 0.5 µg of an oligo-dT primer in a 50 µL total volume. The mixture also contained 0.5 mM of each dNTP (GE-Healthcare), 75 mM KCl, 3 mM MgCl_2_, 10 mM DTT and 0.1 mg/ml Bovine serum albumin in 50 mM Tris-HCl, pH 8.3. The reaction mixture was incubated at 50°C for 60 min and the product was directly used for PCR amplification or stored at −20°C.

### Polymerase chain reaction

Aliquots of 1 µL of crude cDNA were amplified in a Bio-Rad Gene CyclerTM thermocycler, using 2.5 units of *Thermus aquaticus* DNA polymerase (GE-Healthcare), 1 mM of each dNTP (GE-Healthcare), 1 µM of each PCR primer, 50 mM KCl, 2.5 mM MgCl_2_ and 0.1 mg/ml Bovine serum albumin in 10 mM Tris-HCl, pH 8.3, containing 0.1% v/v Triton X-100. At the 5′ end, we used a specific primer corresponding to the sequence encoding the first six amino acids of the mature protein. The primer also contained an Nde I restriction site for ligation into the expression vector and providing at the same time the ATG codon for an additional methionine in position 1. At the 3′ end a specific primer was used, encoding the last six amino acids, followed by a stop codon and an Eco RI restriction site for ligation into the expression vector. Therefore, we used the following primers for the OBP5 (enzyme restriction sites are underlined):

*fw*AgamOBP5 Nde: 5′- AACATATGGCGATGACGCGAAAACAA-3′

*rv*AgamOBP5 Eco: 5′- GTGAATTCTTATTAGGGAAAGAGAAACAC-3′

After a first denaturation step at 95°C for 5 min, we performed 35 amplification cycles (1 min at 95°C, 30 sec at 50°C, 1 min at 72°C) followed by a final step of 7 min at 72°C. An amplification product of about 400 bp, in agreement with the expected size was obtained.

### Cloning and sequencing

The crude PCR product was ligated into a pGEM (Promega) vector without further purification, using a 1∶5 (plasmid∶ insert) molar ratio and incubating the mixture overnight at room temperature. After transformation of *E. coli* XL-1 Blue competent cells with the ligation product, positive colonies were selected by PCR using the plasmid's primers SP6 and T7 and grown in LB/ampicillin medium. DNA was extracted using the Plasmid MiniPrep Kit (Euroclone) and custom sequenced at Eurofins MWG (Ebersberg, Germany).

### Cloning in expression vectors

pGEM plasmid containing the sequence of OBP5 (Acc. No. Q8T6R6) was digested with Nde I and Eco RI restriction enzymes for two hours at 37°C and the digestion product was separated on agarose gel. The obtained fragment was purified from gel using QIAEX II Extraction kit (Qiagen) and ligated into the expression vector pET-5b (Novagen, Darmstadt, Germany), previously linearized with the same enzymes. The resulting plasmid was sequenced and shown to encode the mature protein.

### Preparation of the recombinant protein

For expression of recombinant protein, pET-5b vector containing the sequence of OBP5 was used to transform BL21(DE3)pLysS *E. coli* cells. Protein expression was induced by addition of Isopropyl-1-thio-β-D-galacto-pyranoside to a final concentration of 0.4 mM when the culture had reached a value of O.D._600_ = 0.8. Cells were grown for further 2 hours at 37°C, then harvested by centrifugation and sonicated. After centrifugation, OBP5 was present as inclusion bodies. The pellet from 1 L of culture was solubilised in 10 mL of 8 M urea, 1 mM DTT in 50 mM Tris buffer, pH 7.4, then diluted to 100 mL with Tris buffer and dialysed three times against Tris buffer.

Purification of the protein was accomplished by combinations of chromatographic steps on anion-exchange resins DE-52 (Whatman) and QFF (GE-Healthcare), along with standard protocols previously adopted for other Odorant-Binding Proteins [49], [50].

### Preparation of antisera

Antisera against OBP4 (Acc. no. Q6T6R7) and OBP5 were obtained by injecting rabbits subcutaneously and intramuscularly with 300 µg of recombinant protein, followed by two additional injections of 150 µg after 15 and 30 days. The protein was emulsified with an equal volume of Freund's complete adjuvant for the first injection and incomplete adjuvant for further injections. The animals were bled 10 days after the last injection and the sera were used without further purification. The rabbits were individually housed in large cages, at constant temperature, and all operations were performed according to ethical guidelines to minimize pain and discomfort to animals.

### Western blot experiments

After electrophoretic separation under denaturing conditions (14% SDS-PAGE), duplicate gels were stained with 0.1% Coomassie blue R250 (Euroclone) in 10% acetic acid, 25% ethanol, or electroblotted on a Trans-Blot nitrocellulose membrane (Bio-Rad Lab) by the procedure of Kyhse-Andersen [51]. After treatment with 2% powdered skimmed milk/0.05% Tween 20 in Phosphate Buffer Saline overnight, the membrane was incubated with the crude antiserum against the protein at a dilution of 1∶500 (2 h) and then with goat anti-rabbit IgG horseradish peroxidase conjugate (dilution 1∶1000; 1 h). Immunoreacting bands were detected by treatment with 4-chloro-1-naphthol and hydrogen peroxide.

## Results and Discussion

### Proteomic analysis of antennae

**Figure 1:**
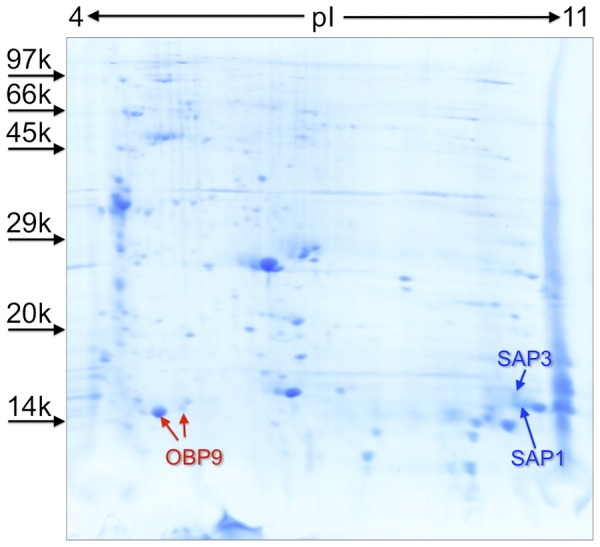
Two-dimensional gel electrophoretic separation of an extract from 1,100 antennae of *An. gambiae*. The gel was stained with colloidal Coomassie Brilliant Blue and all the spots migrating with apparent molecular weight lower than 40: Phosphorylase b, from rabbit muscle (97 kDa), Bovine serum albumin (66 kDa), Ovalbumin (45 kDa), Carbonic anhydrase (29 kDa), Trypsin inhibitor (20 kDa), α-Lactalbumin (14 kDa).

Our first attempt to identify OBPs and CSPs in the antennae of mosquitoes followed a classical approach. The 2D-gel, prepared with the antennae of 1,100 males (Figure 1), produced 79 protein spots in the region of MW lower than 40 kDa, that were excised and analysed. This choice included also proteins longer than classic OBPs, such as C-plus OBPs, salivary OBPs and so-called “tandem OBPs”. However, the only OBP identified in this experiment was OBP9. In addition, two proteins of the CSP family, named SAP1 and SAP3, were detected. These results reasonably exclude the presence of other OBPs and CSPs, at least above the Coomassie staining detection limit. However, we felt that this method could not be sensitive enough to detect all the proteins present in our sample and probably not applicable to the smaller antennae of female mosquitoes. Therefore, we decided to apply a shot-gun approach, that does not require a 2D-gel, but analyses a tryptic digest of a crude protein extract by nano-HPLC and MS/MS. Such technique recently proved to be fast and efficient, requiring at the same time very small biological samples, as in the case of the antennae of *Drosophila* [9], [52].

Applying this method to antennal samples from 600 males and 600 virgin females of *An. gambiae*, we identified 2958 proteins (2605 in females and 2634 in males). A complete list of such proteins is reported in file S1, grouped according to their Pfam descriptors. Pfam PF01395, described as “PBP/GOBP”, includes all OBPs (classic, C-plus- and atypical), while Pfam PF03392, described as “Insect Pheromone-binding” includes CSPs and SAPs.

Most of the identified proteins and corresponding Pfam were common between the two sexes and not differently expressed. Table 1 reports Pfam overexpressed or identified in only one sex. Within the “PBP/GOBP” Pfam, 3 proteins are female specific and 12 are more abundantly expressed than in males. On the other hand, the expression of three CSPs, belonging to the “Insect pheromone-binding” Pfam, was male biased.

**Table 1:** Protein families (Pfam) enriched or expressed in the antennae of females or males (N: number of protein excusive or more expressed in one sex; *P*: Fisher exact test probability).

Female antennae only			
Pfam ID	Description	N	*P* value
PF00005	ABC transporter	1	0.0492
PF00050	Kazal-type serine protease inhibitor domain	1	0.01
PF00095	WAP-type (Whey Acidic Protein) \four-disulfide core\	1	0.01
PF00098	Zinc knuckle	1	0.01
PF00233	3n5n-cyclic nucleotide phosphodiesterase	1	0.0298
PF00454	Phosphatidylinositol 3- and 4-kinase	1	0.0298
PF01395	PBP/GOBP family	3	0.0018
PF01571	Aminomethyltransferase folate-binding domain	1	0.01
PF02872	5n-nucleotidase	1	0.0298
PF04000	Sas10/Utp3/C1D family	1	0.01
PF04968	CHORD	1	0.01
PF06377	Adipokinetic hormone	1	0.02
PF07258	HCaRG protein	1	0.01

Male antennae only			
PFAM ID	Description	N	P value
PF00025	ADP-ribosylation factor family	2	0.0351
PF00082	Subtilase family	1	0.0384
PF00100	Zona pellucida-like domain	2	0.0198
PF00400	WD domain	6	9e-04
PF00432	Prenyltransferase and squalene oxidase repeat	1	0.0384
PF00561	alpha/beta hydrolase fold	2	0.044
PF00575	S1 RNA binding domain	1	0.0384
PF00692	dUTPase	1	0.0384
PF00849	RNA pseudouridylate synthase	1	0.0384
PF01250	Ribosomal protein S6	1	0.0384
PF01380	SIS domain	1	0.0384
PF01426	BAH domain	1	0.0384
PF01556	DnaJ C terminal region	1	0.0384
PF01733	Nucleoside transporter	1	0.0384
PF02146	Sir2 family	1	0.0384
PF02268	Transcription initiation factor IIA	1	0.0384
PF02515	CoA-transferase family III	1	0.0384
PF03083	MtN3/saliva family	1	0.0384
PF03531	Structure-specific recognition protein (SSRP1)	1	0.0384
PF04062	ARP2/3 complex ARPC3 (21 kDa) subunit	1	0.0384
PF04515	Plasma-membrane choline transporter	1	0.0384
PF05018	Protein of unknown function (DUF667)	1	0.0384
PF05172	MPPN (rrm-like) domain	1	0.0384
PF05620	Protein of unknown function (DUF788)	1	0.0384
PF06456	Arfaptin-like domain	1	0.0384
PF06814	Lung seven transmembrane receptor	1	0.0384
PF07159	Protein of unknown function (DUF1394)	1	0.0384
PF08235	LNS2 (Lipin/Ned1/Smp2)	1	0.0384
PF08242	Methyltransferase domain	1	0.0384
PF08799	pre-mRNA processing factor 4 (PRP4) like	1	0.0384
PF09735	Membrane-associated apoptosis protein	1	0.0384
PF10037	Mitochondrial 28S ribosomal protein S27	1	0.0384
PF10211	Axonemal dynein light chain	1	0.0384
PF10270	Membrane magnesium transporter	1	0.0384
PF11069	Protein of unknown function (DUF2870)	1	0.0384
PF11467	Lens epithelium-derived growth factor (LEDGF)	1	0.0384
PF12494	Protein of unknown function (DUF3695)	1	0.0384
PF12612	Tubulin folding cofactor D C terminal	1	0.0384

Enriched in females			
PFAM ID	Description	N	P value
PF00379	Insect cuticle protein	10	0
PF01249	Ribosomal protein S21e	1	0.0133
PF01395	PBP/GOBP family	12	0
PF01413	C-terminal tandem repeated domain in type 4 procollagen	1	0.0393
PF04527	Drosophila Retinin like protein	1	0.0393
PF07993	Male sterility protein	1	0.0393
PF08920	Splicing factor 3B subunit 1	1	0.0133

Enriched in males			
PFAM ID	Description	N	P value
PF00024	PAN domain	1	0.0305
PF00079	Serpin (serine protease inhibitor)	3	0.0049
PF00230	Major intrinsic protein	2	9e-04
PF00293	NUDIX domain	3	5e-04
PF00344	eubacterial secY protein	1	0.0305
PF00379	Insect cuticle protein	5	0.0365
PF00709	Adenylosuccinate synthetase	1	0.0305
PF01007	Inward rectifier potassium channel	1	0.0305
PF01214	Casein kinase II regulatory subunit	1	0.0305
PF01462	Leucine rich repeat N-terminal domain	1	0.0305
PF01652	Eukaryotic initiation factor 4E	1	0.0305
PF01683	EB module	1	0.0305
PF01966	HD domain	1	0.0305
PF02209	Villin headpiece domain	1	0.0305
PF02781	Glucose-6-phosphate dehydrogenase	1	0.0305
PF03022	Major royal jelly protein	2	0.0228
PF03098	Animal haem peroxidase	2	0.0053
PF03148	Tektin family	2	0.0027
PF03392	Insect pheromone-binding family	2	0.0086
PF03870	RNA polymerase Rpb8	1	0.0305
PF04095	Nicotinate phosphoribosyltransferase (NAPRTase) family	1	0.0305
PF05918	Apoptosis inhibitory protein 5 (API5)	1	0.0305
PF06068	TIP49 C-terminus	1	0.0305
PF06602	Myotubularin-related	1	0.0305
PF07479	NAD-dependent glycerol-3-phosphate dehydrogenase C-terminus	1	0.0305
PF09315	Domain of unknown function (DUF1973)	1	0.0305
PF10629	Protein of unknown function (DUF2475)	1	0.0305

Table 2 reports the data relative to the individual 24 OBPs and 4 CSPs identified in the in antennae of both sexes, together with their entry codes and names in Uniprot database. The table also includes three proteins previously reported by Justice and coworkers [53] in the antennae of the same species: the putative antennal carrier protein ANP-1, and two polypeptides named TOL-1 and TOL-2 (TakeOut-Like proteins) considered to be potential carriers for hydrophobic ligands and possibly involved in feeding behaviour. None of these three proteins shows significant sequence similarity with OBPs or CSPs.

**Table 2:** OBPs and CSPs identified in the antennae of *An. gambiae* by shot-gun analysis.

Entry code (*)	Leader protein	Unique peptides (F)	Unique peptides (M)	Sequence coverage % (**)
**Classic Odorant-binding Proteins**				
Q8I8T0 or Q8I8S8 or Q7PLY5	OBP1 or OBP17	13	6	63.9
Q7PLY2	OBP2	4	2	35.7
Q8T6R8	OBP3	9	4	50.8
Q8T6R5	OBP6	1	0	35.7
Q7PXT9; (Q8T6R4)	AgamOBP7	8	2	57.8
Q8I8R2	OBP9	9	4	73.4
F5HMX5; (Q8I8R1)	OBP10	4	1	34.8
Q8I8T5	OBP12	10	4	58.5
Q8I8S7; (Q8I8S6)	AgamOBP18	1	1	16.6
Q7Q9J3 or Q8I8S4	OBP20	6	2	40.8
Q7PGA3; (Q8I8S1)	OBP22	4	0	31.8
Q8I8R7;Q7Q088;Q6J291	AgamOBP25	4	2	40.3
Q8I8R6	AgamOBP26	4	1	38.2
**C-plus Odorant-binding Proteins**				
Q7QCC4	OBPjj9	3	2	16.7
Q7PF80 or Q7YW68	OBP47	6	1	30.7
Q7YW67 or Q8MMI9 or Q6J290	AgamOBP48	7	3	39.5
Q5TYJ0 or Q8I8R3	OBP54	2	0	5.3
Q7Q2W3	OBP57	3	2	15.7
**Salivary Odorant-binding Proteins**				
Q7Q488 (Q9UB30)	D7-related 1 protein	3	0	17.0
Q9UB31 (O76815)	D7-related 2 protein	6	1	50.6
Q9UB32 or Q7Q487 (O76816)	D7-related 3 protein	2	0	15.6
Q7PNF2 or Q9BIH3	D7-related 4 protein	4	0	27.3
SNAP_ANOPHELES00000005748 (Q7Q484; Q7PJ76; Q8WR35)	SNAP_ANOPHELES00000005748	8	3	17.4
Q7PP74	AGAP006278-PA	7	6	27.8
**Chemosensory Proteins**				
Q7Q3U7 or Q8T6R3	Sensory appendage protein SAP-1	8	6	59.1
Q6H8Z3	Sensory appendage protein SAP-2	7	7	46.5
Q6H8Z2	Sensory appendage protein SAP-3	6	7	46.8
Q6H8Y9	chemosensory protein CSP3	5	3	42.9
**Other proteins**				
Q7Q2T1	putative antennal carrier protein ANP-1	4	5	50.0
Q86PT5	Putative antennal carrier protein TOL-1	8	4	37.9
Q7PQP2	Putative antennal carrier protein TOL-2	3	3	40.7

In several cases, entries with the same or similar names refer to very similar sequences, likely originated from different strains of mosquitoes. In the leader protein column we report the name of the sequence with the highest coverage, as reported in the SwissProt database. OBP9 was also identified as the only olfactory protein in eggs, on the basis of two peptides with a coverage of 25.9%. Unique peptides are those characteristic of each sequence. F: females, M: males.

(*) in Swissprot or genome for leader protein (and other proteins in the group).

(**) Total sequence coverage was calculated on the basis of the sum peptides identified in males and females.

In a few cases, because of high identity of sequences, more than one OBP was identified on the basis of the same set of peptides. As an example, OBP1 and OBP17 share the same amino acid sequence, but the latter presents a longer C-terminus (155 vs 144 aa). On the other hand, we could distinguish two proteins Q8T6R5 and Q8I8S7 sharing 97% of their amino acid sequence by the presence of one peptide unique to each of them (Figures S1 and S2).

The identified OBPs can be assigned to three different groups: 13 classical OBPs, 5 C-plus OBPs and 6 salivary OBPs. These latter OBPs, previously reported as belonging to the D7 proteins, are in fact abundant in mosquitoes saliva [54]. Since they are longer than classic OBPs (about 300 amino acids against 120–130) and are characterised by two extra cysteines in addition to the six of the conserved motif, they can also be assigned to the sub-class of C-plus OBPs. A special note deserves SNAP_ANOPHELES00000005748, that is much longer than other D7 proteins. In fact, it contains two typical D7 domains (each with 8 cysteines) connected by a short amino acid bridge. The sequence of the first domain is nearly identical to that of the D7 protein Q7Q484. A function of these salivary proteins in chemical communication has not been investigated, although their presence in the antennae might suggest a role in odorant detection. Of the 4 identified CSPs, 3 have been previously reported in the literature as SAPs [14].

**Figure 2:**
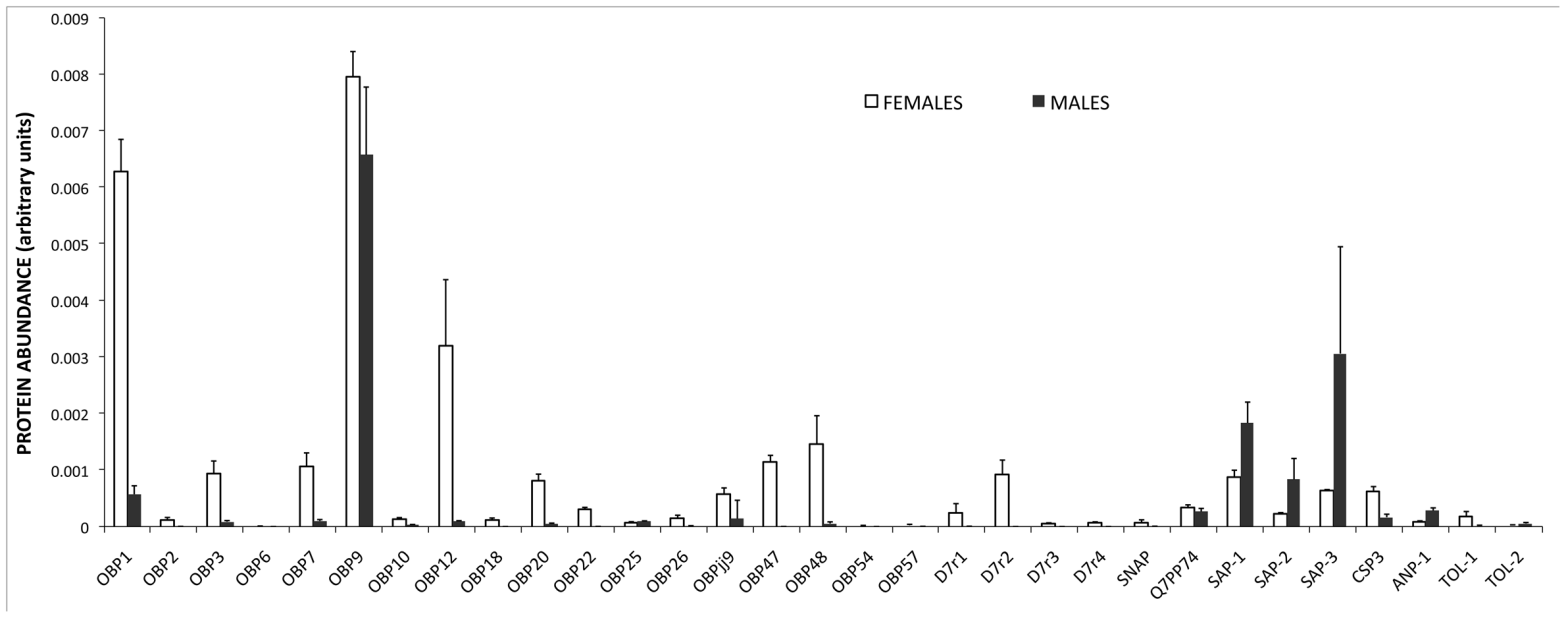
Abundance of OBPs, CSPs and other proteins in the antennae of *An. gambiae* males and females, as reported in Table 1. The evaluation of relative abundance (in arbitrary units) is based on the values produced by MaxQuant (see text). The values are the averages of three sets of analyses. Error bars represent standard error of the mean. By far the most abundant proteins in male antennae are OBP9, SAP1 and SAP3, in agreement with the results of the 2D-gel (Figure 1).

Perhaps the major drawback of the shot-gun method is the difficulty in evaluating the abundance of each protein using label-free approaches [55]. For the evaluation of our results, we have used the protein “Intensity” values as produced by “MaxQuant” software, based on the areas of the peptide peaks in the LC/MS analysis. Three samples of male and three of female antennae were analysed (each one in triplicate) and the areas of peptides were averaged for each protein over the three replicates. These values were then normalised dividing each of them by the total intensity value relative to all the proteins identified. Finally we plotted the averages of the three analyses for males and females, together with their standard errors (Figure 2)

According to the data of Figure 2, the most represented OBPs (including classical and C-plus sequences) in the antennae of females are, in the order, #9, 1/17 and 12, followed by OBPs #48, 47, 7, 3 and 20, that are expressed at lower levels and few others only detectable in traces. D7r2 is the best represented among the salivary proteins. Among the CSPs, we could only detect relatively low levels of the three SAPs and the CSP3. In males, according to the same criterion, the picture is quite different, with OBP9 as the only protein of this family present at high levels, together with SAP1 and SAP3, also strongly represented. This result is in good agreement with 2D-gel data on male antennae, where we could only detect the three above mentioned proteins.

Overall, we can observe that female antennae generally express a larger number and higher quantities of OBPs than males, while the situation is reversed for CSPs.

Our results are in fairly good, but not complete, agreement with a microarray-based RNA analysis [14], which ranked female antennal OBPs in the following decreasing order of abundance: #5, 48, 1, 17, 9, 47, 3, 7, 4 and 20. All these genes, with the exception of OBP4 and OBP5, encode proteins that in our analysis were classified as “abundant” or “well represented”, although not in the same order.

These data are partially confirmed by a more recent a transcriptome analysis [35], that however failed to detect OBP9, a protein found in the present work as the most abundant OBP in all tissues and developmental stages.

The absence of OBP4 and OBP5 in our analysis posed a major problem, also because OBP4 transcript had been reported in our previous work [43], using mosquitoes of the same age and physiological state as those of the present research. In order to clarify this point, we decided to perform Western blot experiments.

### Western blot experiments

**Figure 3:**
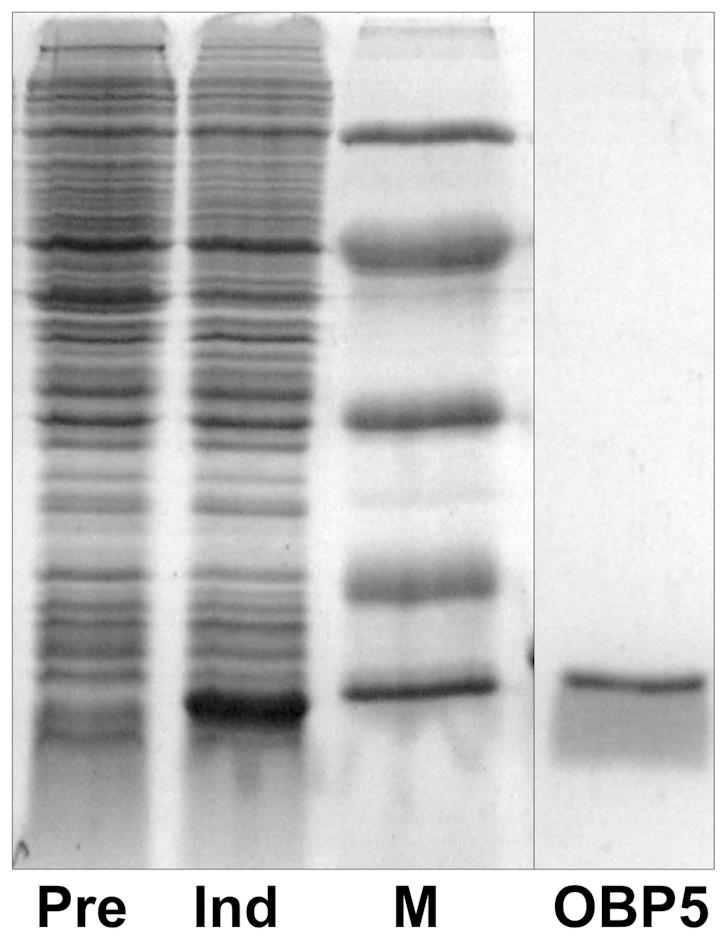
Expression of *An. gambiae* OBP5 in *E. coli*. SDS-PAGE of bacterial pellets before (Pre) and after (Ind) induction of the culture with Isopropyl-1-thio-β-D-galacto-pyranoside. Molecular weight markers are, from the top: Bovine serum albumin (66 kDa), Ovalbumin (45 kDa), Carbonic anhydrase (29 kDa), Trypsin inhibitor (20 kDa), α-Lactalbumin (14 kDa). OBP5: purified protein.

Therefore, we expressed OBP5 in bacteria, adopting the classic procedure utilised for the expression of other OBPs. As most of these proteins, OBP5 was present as inclusion bodies and was solubilised and purified using our standard protocol successfully adopted for many proteins of this class [49], [50] (Figure 3).

**Figure 4:**
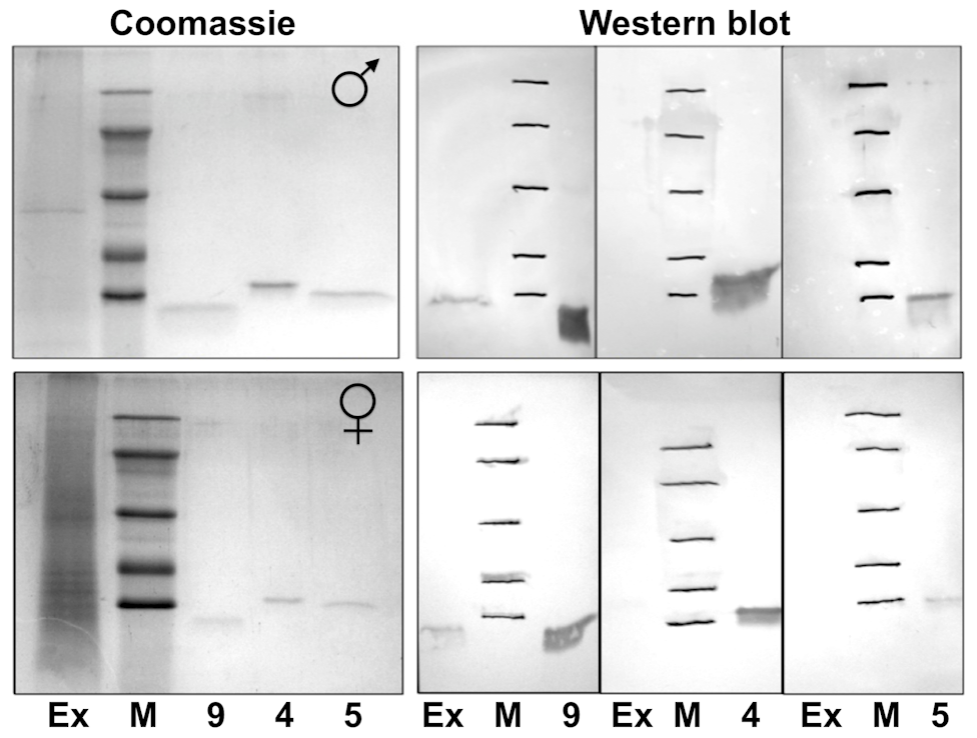
Western-blot of crude antennal extracts of male and female *An. gambiae*, using polyclonal antisera against OBPs 9, 4 and 5. Left panels: SDS-PAGE of crude extracts (Ex) and sample of purified OBPs as indicated by their numbers. Right panels: Western-blot analysis of crude extracts (Ex) performed with the three antisera. A sample of OBPs 9, 4 and 5 (0.5 µg of each protein) utilised for raising the antibodies was also loaded on the same gel. OBP4 and 5 are not detectable in our experimental conditions, while OBP9 is present in both sexes, in agreement with the shotgun experiment results. Molecular weight markers are, from the top: Bovine serum albumin (66 kDa), Ovalbumin (45 kDa), Carbonic anhydrase (29 kDa), Trypsin inhibitor (20 kDa), α-Lactalbumin (14 kDa).

Polyclonal antibodies were raised against the newly produced OBP5 and the previously described OBP4 [43] and used in Western blot experiments on crude extracts of female and male antennae. Figure 4 reports the results of the immunodetection. As controls for the antisera, we included samples of the purified proteins, while an internal control for the extract was provided by OBP9 that had been detected as the most intense spot in the 2D-gel of male antennae (Figure 1) and previously reported in the antennae of both sexes [56]. The expression of OBP9 and the production of a polyclonal antiserum is part of a currently ongoing research (Qiao et al., unpublished). While we could clearly stain OBP9 in the extract, we were not able to get evidence for the presence of OBP4 or OBP5 (Figure 4). We then repeated the Western blot experiments using polyclonal antisera against OBP47 and SAP3, two proteins expressed at lower levels than OBP9, that could provide alternative positive controls. As we failed to stain either of these proteins, both detected in our proteomic study, we concluded that our Western blot method is not sensitive enough for proteins expressed at lower levels, and consequently we cannot exclude the presence of OBP4 and OBP5 in the antennal extracts.

On the other hand, there could be alternative reasons for the absence of OBP4 and OBP5 in our shot-gun experiments, including the possibility that the synthesis of these proteins could be triggered by some physiological events, such as mating or ingesting a blood meal.

### Proteomic analysis on pre-adult stages and eggs

We also decided to investigate the presence of OBPs and CSPs in pre-adult stages and in eggs. Given the relatively large samples available for larvae and pupae, we have chosen to adopt for this study a 2D-gel electrophoresis coupled to mass spectrometry analysis.

**Figure 5:**
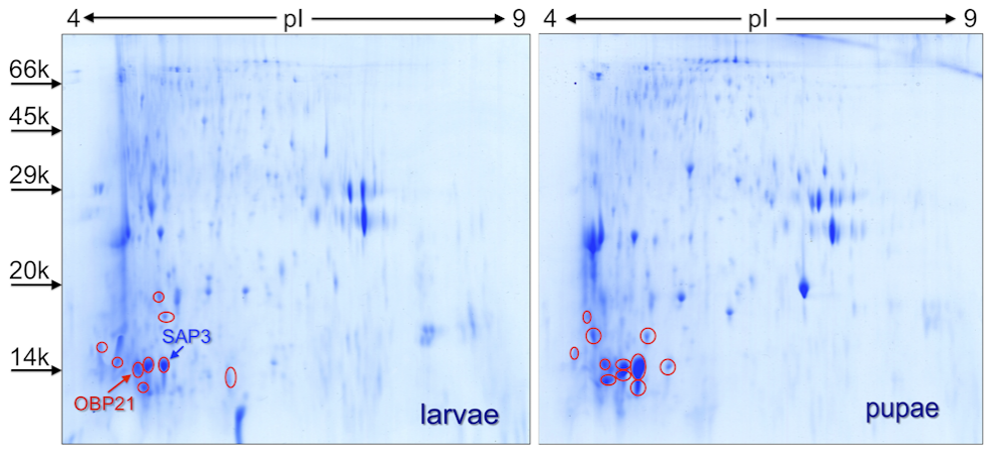
Two-dimensional gel electrophoretic separation of extracts from 100 fourth instar larvae and 100 pupae of *An. gambiae*. The gel was stained with colloidal Coomassie Brilliant Blue and all the spots migrating with apparent molecular weight lower than 24(coverage by aminoacid sequence up to 61.87%), found in several spots (red circles). In larvae we could also detect OBP21 (Entry code in Uniprot Q8I8S3; coverage by aminoacid sequence 9.16%) and SAP3 (coverage by aminoacid sequence up to 18.25%), present in spots where also OBP9 was identified. Molecular weight markers are, from the top: Phosphorylase b, from rabbit muscle (97 kDa), Bovine serum albumin (66 kDa), Ovalbumin (45 kDa), Carbonic anhydrase (29 kDa), Trypsin inhibitor (20 kDa), α-Lactalbumin (14 kDa).

Crude extracts from 100 larvae at 4^th^ instar or 100 pupae of *An. gambiae* were separated on 2D-gels (Figure 5) and the spots analysed as described in the Materials and Methods section. The mass spectrometry analysis performed on the digests of all the spots migrating with apparent molecular masses lower than 24 kDa has revealed the presence of OBP9 as the sole protein of this class, that however appears in several abundant spots (red circles). This phenomenon, that needs to be further investigated, might indicate the occurrence of different forms of OBP9, possibly the products of post-translational modifications. The widespread expression of OBP9 in *An. gambiae* also includes a report of this protein in the hemolymph of adults [57]. In the gel of larvae we could also detect OBP21, a protein absent in the antennae of adults, and SAP3 in spots where also OBP9 was identified.

A sample of 100 eggs was utilised for a shot-gun analysis, as reported in the Materials and Methods section. The only olfactory protein identified was OBP9, whose presence was based on two peptides found in all three replicates, with a coverage of 25.9%.

## Conclusions

The main results of our work can be so summarised:

1. There is a strong sexual dimorphism in the number of OBPs expressed in the antennae. While only a few OBPs can be found in males with only OBP9 expressed at a high level, females are endowed with at least 8 members abundantly expressed, and 14 more that are still clearly detectable. Different expression of OBPs between sexes had been previously reported in *Drosophila melanogaster* [58].
2. Two of the most expressed OBPs (#47 and #48) belong to the C-plus OBPs. In particular, these two proteins contain 4 and 7 cysteines, respectively, in addition to the six of the conserved motif and a more complex structure, recently elucidated for OBP47 [22]. It is not yet clear whether these unusual proteins might be involved in chemodetection like classic OBPs, or else be endowed with alternative functions and modes of action.
3. In pre-adult stages and in eggs the exceptional abundance of OBP9 and the absence of other proteins of the same family suggest that this protein might be involved in functions other than chemoreception. This fact is particularly true for eggs, that are not endowed with chemoreception.
4. The repertoire of OBPs present at detectable levels (13 classical OBPs, 5 C-plus OBPs, 6 salivary OBPs) is much lower than the number of genes encoding such proteins in *An. gambiae*, thus providing a reduced number of molecular targets for further biochemical research and actions aimed at mosquito population control.

## Supporting Information

Figure S1 **Annotated MS/MS spectrum of the peptide QIEILPENYR (m/z = 637.84).** Peptide sequence is unique for the protein Q8I8S7 (OBP18).
(PDF)

Figure S2 **Annotated MS/MS spectrum of the peptide QIEILPETYR (m/z = 631.34).** Peptide sequence is unique for the protein Q8T6R5 (OBP6).
(PDF)

File S1 **Complete list of proteins identified in Anopheles gambiae antennae through the shotgun approach using ANDROMEDA** [**47**] **as search engine.** Column A, protein identity as reported in An. gambiae genome and in UniProKB; Column B, identity of leader protein within the protein group; Column C and F, protein descriptio in the genome and in UniprotKB; Column D, protein family; Column E, protein family description; Column G, molecular weight of leader protein; Colum H, protein posterior error probability.
(XLSX)

## References

[1] Snow RW, Guerra CA, Noor AM, Myint HY, Hay SI (2005) The global distribution of clinical episodes of Plasmodium falciparum malaria. Nature 434: 214–217. 15759000 10.1038/nature03342 PMC3128492

[2] Turner SL, Li N, Guda T, Githure J, Cardé RT, et al (2001) Ultra-prolonged activation of CO2-sensing neurons disorients mosquitoes. Nature 474: 87–91. 10.1038/nature10081 PMC3150595 21637258

[3] Corbel V, Stankiewicz M, Pennetier C, Fournier D, Stojan J, et al (2009) Evidence for inhibition of cholinesterases in insect and mammalian nervous systems by the insect repellent deet. BMC Biology 7: 47. 19656357 10.1186/1741-7007-7-47 PMC2739159

[4] Pelosi P, Zhou J-J, Ban LP, Calvello M (2006) Soluble proteins in insect chemical communication. Cell Mol Life Sci 631: 658–1676. 10.1007/s00018-005-5607-0 PMC11136032 16786224

[5] Xu P, Atkinson R, Jones DN, Smith DP (2005) *Drosophila* OBP LUSH is required for activity of pheromone-sensitive neurons. Neuron 45: 193–200. 15664171 10.1016/j.neuron.2004.12.031

[6] Grosse-Wilde E, Svatos A, Krieger J (2006) A pheromone-binding protein mediates the bombykol-induced activation of a pheromone receptor in vitro. Chem Senses 31: 547–55. 16679489 10.1093/chemse/bjj059

[7] Matsuo T, Sugaya S, Yasukawa J, Aigaki T, Fuyama Y (2007) Odorant-Binding Proteins OBP57d and OBP57e affect taste perception and host-plant preference in *Drosophila sechellia* . PLoS Biol 5: e118. 17456006 10.1371/journal.pbio.0050118 PMC1854911

[8] Laughlin JD, Ha TS, Jones DNM, Smith DP (2008) Activation of pheromone-sensitive neurons is mediate by conformational activation of Pheromone-binding protein. Cell 133: 1255–1265. 18585358 10.1016/j.cell.2008.04.046 PMC4397981

[9] Swarup S, Williams TI, Anholt RR (2011) Functional dissection of Odorant binding protein genes in *Drosophila melanogaster* . Genes Brain Behav 10: 648–657. 21605338 10.1111/j.1601-183X.2011.00704.x PMC3150612

[10] Sun YF, De Biasio F, Qiao HL, Iovinella I, Yang SX, et al (2012) Two Odorant-Binding Proteins Mediate the Behavioural Response of Aphids to the Alarm Pheromone (*E*)-ß-Farnesene and Structural Analogues. PLoS ONE 7: e32759. 22427877 10.1371/journal.pone.0032759 PMC3299684

[11] Holt RA, Subramanian GM, Halpern A, Sutton GG, Charlab R, et al (2002) The genome sequence of the malaria mosquito *Anopheles gambiae* . Science 298: 129–149. 12364791 10.1126/science.1076181

[12] Fox AN, Pitts RJ, Robertson HM, Carlson JR, Zwiebel LJ (2001) Candidate odorant receptors from the malaria vector mosquito *Anopheles gambiae* and evidence of down-regulation in response to blood feeding. Proc Natl Acad Sci U S A 98: 14693–14697. 11724964 10.1073/pnas.261432998 PMC64743

[13] Hill CA, Fox AN, Pitts RJ, Kent LB, Tan PL, et al (2002) G protein-coupled receptors in *Anopheles gambiae* . Science 298: 176–178. 12364795 10.1126/science.1076196

[14] Biessmann H, Nguyen QK, Le D, Walter MF (2005) Microarray-based survey of a subset of putative olfactory genes in the mosquito *Anopheles gambiae* . Insect Mol Biol 14: 575–589. 16313558 10.1111/j.1365-2583.2005.00590.x

[15] Carey AF, Wang G, Su CY, Zwiebel LJ, Carlson JR (2010) Odorant reception in the malaria mosquito *Anopheles gambiae* . Nature 464: 66–72. 20130575 10.1038/nature08834 PMC2833235

[16] Wang G, Carey AF, Carlson JR, Zwiebel LJ (2010) Molecular basis of odor coding in the malaria vector mosquito *Anopheles gambiae* . Proc Natl Acad Sci USA 107: 4418–4423. 20160092 10.1073/pnas.0913392107 PMC2840125

[17] Sun Y-L, Huang L-Q, Pelosi P, Wang C-Z (2012) Expression in Antennae and Reproductive Organs Suggests a Dual Role of an Odorant-Binding Protein in Two Sibling *Helicoverpa* Species. PLoS ONE 7: e30040. 22291900 10.1371/journal.pone.0030040 PMC3264552

[18] Calvo E, Mans BJ, Ribeiro JM, Andersen JF (2009) Multifunctionality and mechanism of ligand binding in a mosquito antiinflammatory protein. Proc Natl Acad Sci U S A 106: 3728–3733. 19234127 10.1073/pnas.0813190106 PMC2656148

[19] Zhou JJ, He XL, Pickett JA, Field LM (2008) Identification of odorant-binding proteins of the yellow fever mosquito *Aedes aegypti*: genome annotation and comparative analyses. Insect Mol Biol 17: 147–163. 18353104 10.1111/j.1365-2583.2007.00789.x

[20] Scaloni A, Monti M, Angeli S, Pelosi P (1999) Structural analyses and disulfide-bridge pairing of two odorant binding proteins from *Bombyx mori* . Biochem Biophys Res Commun 266: 386–391. 10600513 10.1006/bbrc.1999.1791

[21] Leal WS, Nikonova L, Peng G (1999) Disulfide structure of the pheromone binding protein from the silkworm moth, *Bombyx mori* . FEBS Lett 464: 85–90. 10611489 10.1016/s0014-5793(99)01683-x

[22] Lagarde A, Spinelli S, Qiao H, Tegoni M, Pelosi P, et al (2011) Crystal structure of a novel type of odorant binding protein from *Anopheles gambiae*, belonging to the C+ class. Biochem J 437: 423–430. 21561433 10.1042/BJ20110522

[23] Wanner KW, Willis LG, Theilmann DA, Isman MB, Feng Q, et al (2004) Analysis of the insect OS-D-like gene family. J Chem Ecol 30: 889–911. 15274438 10.1023/b:joec.0000028457.51147.d4

[24] Picimbon JF (2003) Biochemistry and Evolution of OBP and CSP proteins. In: Blomquist GJ, Vogt RG (eds) Insect Pheromone Biochemistry and Molecular Biology, Elsevier Academic Press, London, pp 539–566.

[25] Jacquin-Joly E, Vogt RG, Francois MC, Nagnan-Le Meillour P (2001) Functional and expression pattern analysis of chemosensory proteins expressed in antennae and pheromonal gland of *Mamestra brassicae* . Chem Senses 26: 833–844. 11555479 10.1093/chemse/26.7.833

[26] Maleszka J, Forêt S, Saint R, Maleszka R (2007) RNAi-induced phenotypes suggest a novel role for a chemosensory protein CSP5 in the development of embryonic integument in the honeybee (*Apis mellifera*). Dev Genes Evol 217: 189–196. 17216269 10.1007/s00427-006-0127-y

[27] Nomura A, Kawasaki K, Kubo T, Natori S (1992) Purification and localization of p10, a novel protein that increases in nymphal regenerating legs of *Periplaneta americana* American cockroach. Int J Dev Biol 36: 391–398. 1445782

[28] Kitabayashi AN, Arai T, Kubo T, Natori S (1998) Molecular cloning of cDNA for p10, a novel protein that increases in the regenerating legs of *Periplaneta americana* (American cockroach). Insect Biochem Mol Biol 28: 785–790. 9807224 10.1016/s0965-1748(98)00058-7

[29] Dyanov HM, Dzitoeva SG (1995) Method for attachment of microscopic preparations on glass for in situ hybridization, PRINS and in situ PCR studies. Biotechniques 18: 822–826. 7619487

[30] Dani FR, Michelucci E, Francese S, Mastrobuoni G, Cappellozza S, et al (2011) Odorant-binding proteins and Chemosensory proteins in pheromone detection and release in the silkmoth *Bombyx mori* . Chem Senses 36: 335–344. 21220518 10.1093/chemse/bjq137

[31] Zhou XH, Ban LP, Iovinella I, Zhao LJ, Gao Q, et al (2012) Diversity, abundance and sex-specific expression of chemosensory proteins in the reproductive organs of the locust *Locusta migratoria manilensis* . Biol Chem 394: 43–54. 10.1515/hsz-2012-0114 23096575

[32] Angeli S, Ceron F, Scaloni A, Monti M, Monteforti G, et al (1999) Purification, structural characterization, cloning and immunocytochemical localization of chemoreception proteins from *Schistocerca gregaria* . Eur J Biochem 262: 745–754. 10411636 10.1046/j.1432-1327.1999.00438.x

[33] Biessmann H, Walter MF, Dimitratos S, Woods DF (2002) Isolation of cDNA clones encoding putative odorant binding proteins from the antennae of the malaria-transmitting mosquito, *Anopheles gambiae* . Insect Mol Biol 11: 123–132. 11966877 10.1046/j.1365-2583.2002.00316.x

[34] Iovinella I, Bozza F, Caputo B, Della Torre A, Pelosi P (2013) Ligand-binding study of *Anopheles gambiae* chemosensory proteins. Chem Senses 38: 409–19. 23599217 10.1093/chemse/bjt012

[35] Pitts RJ, Rinker DC, Jones PL, Rokas A, Zwiebel LJ (2011) Transcriptome profiling of chemosensory appendages in the malaria vector *Anopheles gambiae* reveals tissue- and sex-specific signatures of odor coding. Genomics 12: 271–288. 21619637 10.1186/1471-2164-12-271 PMC3126782

[36] Amenya DA, Chou W, Li J, Yan GY, Gershon PD, et al (2010) Proteomics reveals novel components of the *Anopheles gambiae* eggshell. J Ins Physol 56: 1414–1419. 10.1016/j.jinsphys.2010.04.013 PMC2918668 20433845

[37] Wogulis M, Morgan T, Ishida Y, Leal WS, Wilson DK (2006) The crystal structure of an odorant binding protein from *Anopheles gambiae*: evidence for a common ligand release mechanism. Biochem Biophys Res Commun 339: 157–164. 16300742 10.1016/j.bbrc.2005.10.191

[38] Ren H, Yang G, Winberg G, Turin L, Mershin A, et al (2009) The crystal structure of an *Anopheles gambiae* odorant-binding protein agamobp22a and complexes with bound odorants. direct submission PDB ID 3QME.

[39] Lagarde A, Spinelli S, Tegoni M, He X, Field L, et al (2011) The crystal structure of odorant binding protein 7 from *Anopheles gambiae* exhibits an outstanding adaptability of its binding site. J Mol Biol 414 (3) 401–12 10.1016/j.jmb.2011.10.005 22019737

[40] Tsitsanou KE, Thireou T, Drakou CE, Koussis K, Keramioti MV, et al (2012) *Anopheles gambiae* odorant binding protein crystal complex with the synthetic repellent DEET: implications for structure-based design of novel mosquito repellents. Cell Mol Life Sci 69: 283–297. 21671117 10.1007/s00018-011-0745-z PMC11114729

[41] Ziemba BP, Murphy EJ, Edlin HT, Jones DN (2013) A novel mechanism of ligand binding and release in the odorant binding protein 20 from the malaria mosquito *Anopheles gambiae* . Protein Sci 22: 11–21. 23081820 10.1002/pro.2179 PMC3575856

[42] Davrazou F, Dong E, Murphy EJ, Johnson HT, Jones DN (2011) New insights into the mechanism of odorant detection by the malaria-transmitting mosquito *Anopheles gambiae* . J Biol Chem 286: 34175–83. 21816826 10.1074/jbc.M111.274712 PMC3190798

[43] Qiao H, He XL, Schymura D, Ban L, Field L, et al (2011) Cooperative interactions between Odorant-Binding Proteins of *Anopheles gambiae* . Cell Mol Life Sci 68: 1799–813. 20957509 10.1007/s00018-010-0539-8 PMC11114539

[44] Della Torre A, Fanello C, Akogbeto M, Dossou-yovo J, Favia G, et al (2001) Molecular evidence of incipient speciation within *Anopheles gambiae* s.s. in West Africa. Insect Mol Biol 10: 9–18. 11240632 10.1046/j.1365-2583.2001.00235.x

[45] Iovinella I, Felicioli A, Niccolini A, Dani FR, Michelucci E, et al (2011) Odorant-Binding Proteins in the mandibular glands of the honeybee as putative carriers of semiochemicals. J Proteome Res 10: 3439–3449. 21707107 10.1021/pr2000754

[46] Rappsilber J, Mann M, Ishihama Y (2007) Protocol for micro-purification, enrichment, pre-fractionation and storage of peptides for proteomics using StageTips. Nat Protoc 2: 1896–906. 17703201 10.1038/nprot.2007.261

[47] Cox J, Neuhauser N, Michalski A, Scheltema RA, Olsen JV, et al (2011) Andromeda: a peptide search engine integrated into the MaxQuant environment. J Proteome Res 10: 1794–805. 21254760 10.1021/pr101065j

[48] Punta M, Coggill PC, Eberhardt RY, Mistry J, Tate J, et al (2012) The Pfam protein families database. Nucleic Acids Research 40: D290–D301. 22127870 10.1093/nar/gkr1065 PMC3245129

[49] Ban LP, Scaloni A, Brandazza A, Angeli S, Zhang L, et al (2003) Chemosensory proteins of *Locusta migratoria* . Insect Mol Biol 12: 125–134. 12653934 10.1046/j.1365-2583.2003.00394.x

[50] Calvello M, Guerra N, Brandazza A, D'Ambrosio C, Scaloni A, et al (2003) Soluble proteins of chemical communication in the social wasp *Polistes dominulus* . Cell Mol Life Sci 60: 1933–1943. 14523553 10.1007/s00018-003-3186-5 PMC11138633

[51] Kyhse-Andersen J (1984) Electroblotting of multiple gels: a simple apparatus without buffer tank for rapid transfer of proteins from polyacrylamide to nitrocellulose. J Biochem Biophys Methods 10: 203–209. 6530509 10.1016/0165-022x(84)90040-x

[52] Anholt RR, Williams TI (2010) The soluble proteome of the *Drosophila* antenna. Chem Senses 35: 21–30. 19917591 10.1093/chemse/bjp073 PMC2795394

[53] Justice R, Dimitratos S, Walter MF, Woods DF, Biessmann H (2003) Sexual dimorphism of antennal gene expression in the malaria vector *Anopheles gambiae* . Insect Mol Biol 12: 581–594. 14986919 10.1046/j.1365-2583.2003.00443.x

[54] Kalume DE, Okulate M, Zhong J, Reddy R, Suresh S, et al (2005) A proteomic analysis of salivary glands of female *Anopheles gambiae* mosquito. Proteomics 5: 3765–3777. 16127729 10.1002/pmic.200401210

[55] Zhu W, Smith JW, Huang CM (2010) Mass Spectrometry-based Label-Free Quantitative Proteomics. J Biomed Biotechnol 10.1155/2010/840518 PMC2775274 19911078

[56] Dani FR, Francese S, Mastrobuoni G, Felicioli A, Caputo B, et al (2008) Exploring Proteins in *Anopheles gambiae* Male and Female Antennae through MALDI Mass Spectrometry Profiling. PLOSone 3: e2822 10.1371/journal.pone.0002822 PMC2474704 18665262

[57] Paskewitz SM, Shi L (2005) The hemolymph proteome of *Anopheles gambiae* . Insect Biochem Mol Biol 35: 815–824. 15944078 10.1016/j.ibmb.2005.03.002

[58] Zhou S, Stone EA, Mackay TF, Anholt RR (2009) Plasticity of the chemoreceptor repertoire in *Drosophila melanogaster* . PLoS Genet 5 (10) e1000681. 19816562 10.1371/journal.pgen.1000681 PMC2750752

