# Supplementary figures and images for: A Proteomic Investigation of Soluble Olfactory Proteins in Anopheles gambiae
Source: PLoS One. 2013 Nov 25;8(11):e75162. doi: 10.1371/journal.pone.0075162 (PMC3839933; doi:10.1371/journal.pone.0075162)

Source: Toni\_20100503\_GM\_Agam\_F\_01  
Scannumber: 7706  
Protein: Q8I8S7  
Peptide Score: 116.52  
Method: ITMS; CID; 1

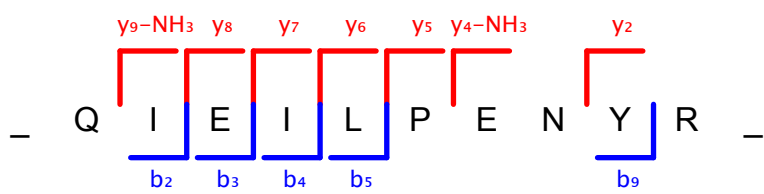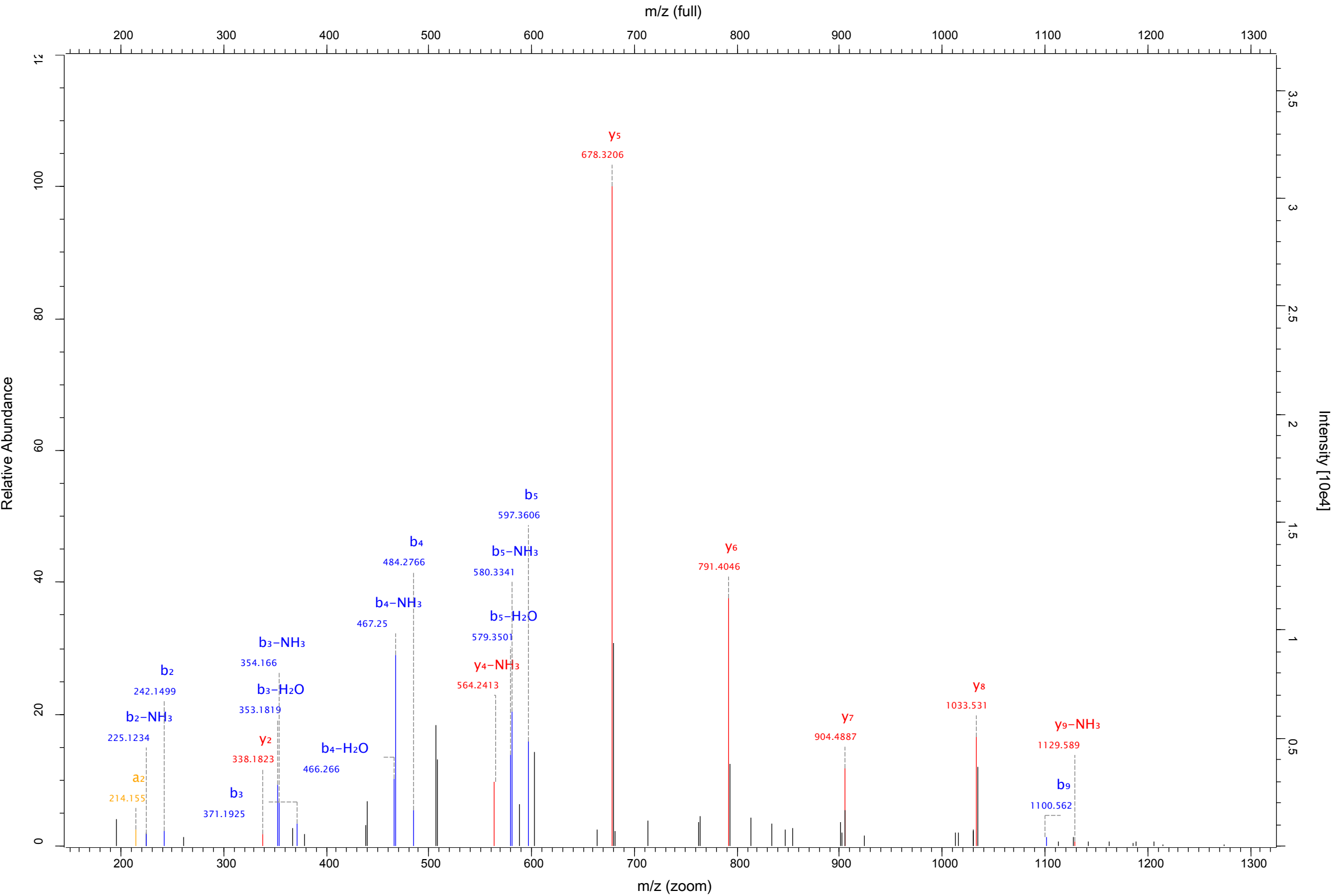

Supplement: Figure S1 — Annotated MS/MS spectrum of the peptide QIEILPENYR (m/z = 637.84). Peptide sequence is unique for the protein Q8I8S7 (OBP18). (PDF) [file pone.0075162.s001.pdf]

Source: Toni\_20100503\_GM\_Agam\_F\_01  
Scannumber: 7932  
Protein: Q8T6R5  
Peptide Score: 91.87  
Method: ITMS; CID; 1

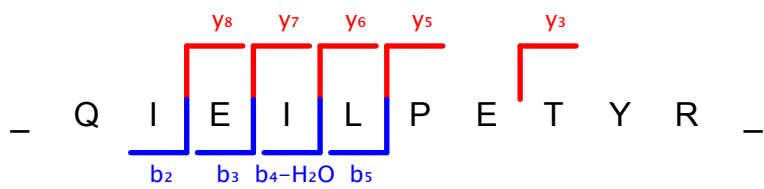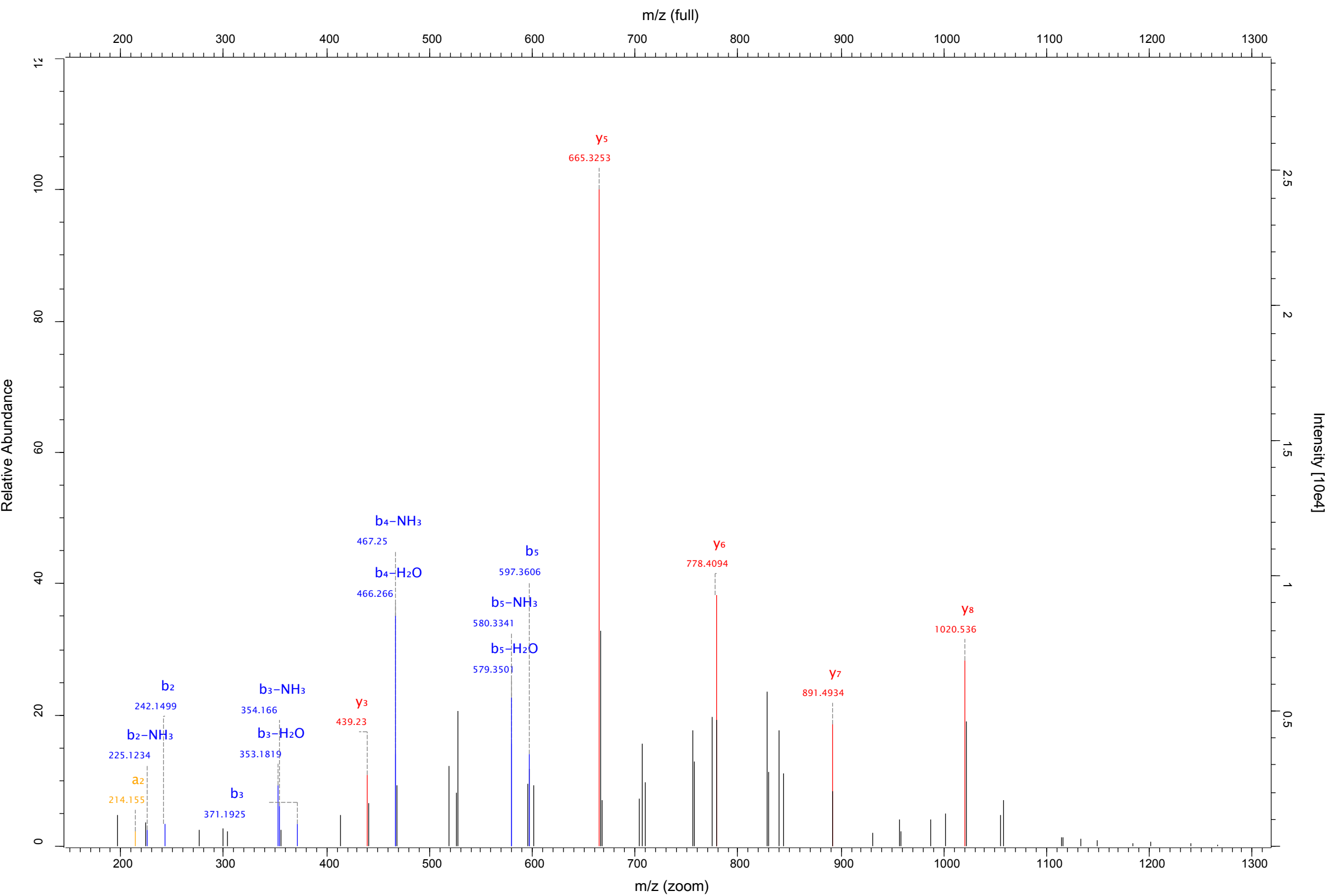

Supplement: Figure S2 — Annotated MS/MS spectrum of the peptide QIEILPETYR (m/z = 631.34). Peptide sequence is unique for the protein Q8T6R5 (OBP6). (PDF) [file pone.0075162.s002.pdf]
